# From Regeneration Failure to Functional Restoration: Unlocking the Neuronal‐Intrinsic Regenerative Capacity as a Therapeutic Frontier for Optic Neuropathy and Glaucoma

**DOI:** 10.1002/cns.70800

**Published:** 2026-02-20

**Authors:** Emma Beard, Safa El‐Bushra, Zahra Kader, Yujiao Jennifer Sun, Ngan Pan Bennett Au

**Affiliations:** ^1^ School of Medicine, Pharmacy and Biomedical Science, Faculty of Science and Health University of Portsmouth Portsmouth UK; ^2^ UCL Institute of Ophthalmology London UK; ^3^ Department of Comparative Biomedical Sciences, School of Veterinary Medicine University of Surrey Guildford UK

**Keywords:** glaucoma, mitochondria‐targeted therapy, optic nerve injury, optic neuropathy, regenerative medicine, small molecules‐based treatment

## Abstract

**Background:**

Optic neuropathy encompasses ocular conditions arising from traumatic or nontraumatic damage to optic nerves, causing permanent visual impairment due to retinal ganglion cell (RGC) loss and disrupted axonal connections. Glaucomatous optic neuropathy represents the most prevalent form, affecting over 70 million individuals worldwide and causing blindness in nearly 4 million people.

**Current Limitations:**

Current treatments targeting elevated intraocular pressure can slow disease progression but cannot restore vision once RGC axons are lost, largely due to regeneration failure in surviving RGCs. This regenerative failure stems not merely from the presence of growth‐inhibitory extrinsic factors, but crucially from diminished neuronal‐intrinsic regenerative capacity in mature RGCs.

**Mechanistic Insights and Therapeutic Implications:**

This review explores how intrinsic growth barriers and inadequate activation of pro‐regenerative genes impede axonal regrowth following injury, and how manipulations of these pathways facilitate axon regeneration and visual recovery in experimental models. Emerging findings suggest that these pro‐regenerative molecules capable of modifying the neuronal‐intrinsic regenerative capacity of RGCs can also preserve visual function in pre‐clinical glaucoma models. Finally, we discuss challenges and future directions for translating these findings into therapies, including gene delivery strategies, remyelination therapeutics, and systems biology‐based in silico drug screening approaches aiming to reshape the therapeutic landscape towards regenerative interventions for glaucomatous and other optic neuropathies.

## Graphical abstract

Optic neuropathy and glaucoma cause permanent visual deficits via retinal ganglion cell (RGC) loss and axon degeneration, affecting millions worldwide. Current treatments slow disease progression but cannot restore lost vision due to regeneration failure in adult RGCs. Understanding underlying molecular mechanisms opens avenues for novel therapies targeting pro‐regenerative pathways to rebuild visual circuitry and improve vision, representing a paradigm shift towards regenerative medicine.

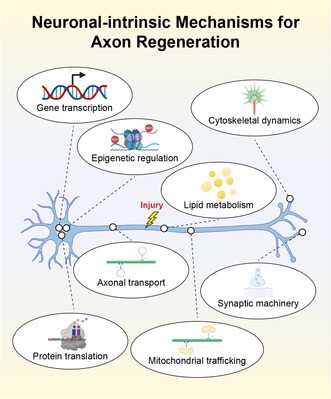

## 1 Introduction

**FIGURE 1:**
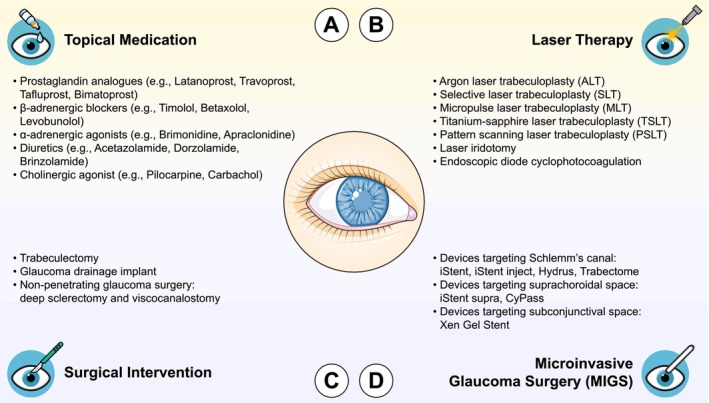
Current treatment options for glaucoma. (A) Topical medications are typically the first‐line therapeutic approach for glaucoma management. A wide range of eye drops has been developed over the years; all aimed at reducing intraocular pressure (IOP). These drugs can be categorized into five major classes: Prostaglandin analogues, β‐adrenergic blockers, α‐adrenergic agonists, diuretics (particularly carbonic anhydrase inhibitors), and cholinergic agonists. (B) Laser therapies are generally considered when topical medications fail to achieve adequate IOP reduction. Common procedures include laser trabeculoplasty, laser iridotomy, and cyclophotocoagulation (especially endoscopic diode cyclophotocoagulation). Various modalities of laser trabeculoplasty have emerged over recent years: Argon laser trabeculoplasty (ALT), selective laser trabeculoplasty (SLT), micropulse laser trabeculoplasty (MLT), titanium‐sapphire laser trabeculoplasty (TSLT), and pattern scanning laser trabeculoplasty (PSLT). (C) Surgical interventions may be warranted when first‐line topical medications and laser therapies cannot effectively control IOP in glaucoma patients. Two established surgical procedures are commonly employed: Trabeculectomy and glaucoma drainage implantation. More recently, non‐penetrating glaucoma surgeries, such as deep sclerectomy and viscocanalostomy, have emerged as alternatives to conventional trabeculectomy, offering reduced complication rates. (D) Microinvasive glaucoma surgery (MIGS) encompasses a family of newer procedures that employ microsurgical instruments or devices to enhance aqueous humor outflow by targeting Schlemm's canal, the suprachoroidal space, or the subconjunctival space and reduce IOP. MIGS procedures are often performed in conjunction with cataract surgery.

Optic neuropathies refer to a group of eye disorders resulting from traumatic or nontraumatic damage to the optic nerve, frequently leading to degeneration of retinal ganglion cell (RGC) axons and progressive RGC loss [1]. These conditions typically result in reduced visual acuity and visual field abnormalities in the affected eye [2]. Among these, glaucomatous optic neuropathy represents the most prevalent form of optic neuropathy [3] and remain the second leading cause of blindness worldwide primarily attributed to elevated intraocular pressure (IOP) [4]. Currently, at least 76 million individuals are affected by glaucoma globally, a figure projected to rise to 111.8 million by 2040 [5]. Within this population, approximately 3.61 million people are estimated to be blind [6]. In the United Kingdom, glaucoma affects over 1 million people [7, 8], with a projected 44% rise in patient numbers by 2035, partly due to a rapid increase in aged population [9]. In the United States, at least 4.22 million individuals are affected by glaucoma, with Black individuals disproportionately impacted [10]. More concerningly, over 90% of glaucoma cases remain undiagnosed in low‐ and middle‐income countries [11]. In contrast to cataract, the leading cause of blindness and typically reversible through surgical interventions, the visual impairment associated with glaucomatous optic neuropathy is often permanent and irreversible, owing to the failure of adult RGC axons to spontaneously regenerate following glaucomatous neurodegeneration. Current therapeutic strategies for glaucoma, including topical medications (e.g., prostaglandin analogues, β‐adrenergic blockers, α‐adrenergic agonists, diuretics, and cholinergic agonists) (Figure 1A), laser therapy (Figure 1B), and surgical interventions (Figure 1C) including microinvasive glaucoma surgery (Figure 1D), are all aimed at lowering IOP to slow or halt disease progression [12, 13]. However, a recent multicentre, placebo‐controlled clinical trial conducted by the United Kingdom Glaucoma Treatment Study (UKGTS) reported that while IOP‐lowering therapies can effectively slow disease progression, they neither reverse nor improve the visual field deficits induced by glaucoma [14]. This underscores the urgent need for alternative therapeutic approaches to restore visual function caused by glaucomatous neurodegeneration, with potential applications extending to other forms of optic neuropathy.

The limited regenerative capacity of mature neurons in the central nervous system (CNS), including RGCs, has remained a fundamental challenge in neuroscience for more than a century. “Once the development was ended, the founts of growth and regeneration of the axons and dendrites dried up irrevocably. In the adult centres, the nerve paths are something fixed, ended, and immutable. Everything may die, nothing may be regenerated. It is for the science of the future to change, if possible, this harsh decree.” This iconic statement by Santiago Ramón y Cajal [15] established the central dogma that shaped regenerative neuroscience for many decades. It was not until the 1980s that Aguayo and colleagues challenged this view, demonstrating that injured axons in the adult CNS could regenerate over long distances when provided with peripheral nerve grafts whilst failing to grow back into CNS tissues [16, 17, 18]. Their work revealed a critical dichotomy: while tissues in the peripheral nervous system (PNS) are inherently growth‐permissive, CNS tissues are predominantly growth‐inhibitory.

Like other tissues, injury typically triggers a conserved repair process involving inflammation, tissue reformation, and remodeling at the lesion. In the CNS, this process leads to the formation of a glial scar, which has traditionally been considered a major growth barrier to spontaneous axon regeneration across the lesion core [19]. Despite numerous efforts to alter the composition of these scar tissues, most studies suggested that this approach failed to elicit long‐distance axon regeneration sufficient to achieve meaningful functional recovery following CNS injuries [20, 21, 22]. A groundbreaking study from the Woolf lab offered a different perspective, showing that a “pre‐conditioning” injury to the peripheral nerve could reprogram sensory neurons (i.e., dorsal root ganglion (DRG) neurons) into an active growth‐competent state. Remarkably, this “pre‐conditioned” peripheral nerve lesion enabled robust axon regeneration into CNS tissues following axotomy to the central nerve without the support of growth‐permissive peripheral nerve grafts [23]. This discovery paved the way for the identification of many regeneration‐associated genes (RAGs) in the following years, including Gap43, Cap23 [24], Sprr1a [25], Atf3 [26], Jun [27], Stat3 [28], and Hspb1 [29, 30] (detailed reviewed in [31, 32]). While manipulation of some RAGs has been shown to enhance axon regeneration and promote functional recovery after peripheral nerve injuries [29, 30, 33], attempts to replicate the “pre‐conditioning” effects using the same strategy to promote CNS regeneration have met with limited success: overexpression of individual RAGs alone has not been sufficient to drive robust axon regeneration following CNS injuries [24, 26].

Over the past two decades, advances in molecular research tools, including high‐throughput sequencing and efficient, neuronal‐specific gene delivery systems for gain‐ or loss‐of‐function assessments, have facilitated the identification of key regulators capable of reverting injured CNS neurons to a growth‐competent state. In this review, we summarize our current understanding of the molecular mechanisms underpinning regeneration failure in the adult CNS and explore how the removal of neuronal‐intrinsic growth barriers and/or the restoration of essential regulators for axonal outgrowth support long‐distance optic nerve regeneration and visual functional recovery following injury. Importantly, several recent strategies that stimulate robust axon regeneration have also demonstrated promising therapeutic potential in preserving visual function within experimental glaucoma models. Finally, we discuss how these pre‐clinical findings might translate into clinically viable strategies as next‐generation regenerative medicines to treat patients with glaucoma and other optic neuropathies.

## 2 Regeneration Failure in the Adult Mammalian CNS Is the Key Obstacle to Functional Recovery

Similar to many CNS neurons, injured RGCs in adult mammals, including rodents such as rats and mice as well as humans, lack the ability to spontaneously regenerate their axons following optic nerve damage. The intrinsic growth ability of RGCs declined dramatically after birth as a result of signals from the amacrine cells in the mature retinae [34]. More critically, significant RGC loss begins within days of injury in mammalian models of optic nerve injury, such as optic nerve crush (ONC) or transection [1, 35]. The total number of RGCs declines significantly to approximately 20% of baseline levels within two weeks post‐ONC compared to uninjured retinae [36, 37, 38]. Despite the poor intrinsic regenerative capacity of adult mammalian RGCs, severed RGC axons can regenerate into growth‐permissive peripheral nerve grafts following transection [39]. Strategies aiming to counteract the inhibitory effects of glial scarring and myelin could enable some extent of axon regeneration [40, 41]. Such strategies might sustain long‐distance optic nerve regeneration and target reinnervation primarily in postnatal mice, where injured RGCs possessed a higher intrinsic regenerative potential and shorter optic tracts [41]. In adult RGCs, which have reduced regenerative capacity, axonal regrowth was restricted to relatively short distances [40].

Lens injury or intravitreal injection of zymosan to the injured eye have shown promise in promoting long‐distance axon regeneration post‐ONC. These interventions induced robust glial responses, characterized by the activation of resident retinal microglia, astrocytes, and Müller cells, as well as the infiltration of peripheral myeloid cells into the injured retinae [1]. This inflammatory milieu stimulated the production of multiple pro‐regenerative factors by Müller cells and immune cells, including oncomodulin, stromal cell‐derived factor 1 (SDF‐1), and CCL5 [42, 43, 44, 45] (The underlying mechanisms of inflammation‐induced pro‐regenerative effects have been extensively discussed in our recent review published elsewhere [1]). Remarkably, such inflammation‐induced responses facilitated RGC axons to regenerate across the full length of the optic nerve following ONC [46]. Although these interventions achieved substantial axon regeneration, leading to the restoration of some visual function, prolonged inflammation led to complications such as cataracts in many treated mice [47]. This poses a major obstacle to their therapeutic application for optic nerve injuries and limits their clinical translatability.

## 3 Unlocking the Neuronal Regenerative Potential by Removing the Intrinsic Growth Barriers

Unlike injured PNS neurons, CNS neurons fail to initiate a robust pro‐regenerative transcriptomic programme, thereby impeding their ability to spontaneously regenerate their damaged axons following axotomy. This regeneration failure is partially attributed to a markedly reduced calcium influx observed in injured RGCs following ONC [48], which is crucial for the initiation of retrograde injury signals for the activation of histone acetylases and RAGs [49]. Additionally, the capacity for local protein synthesis, critical for axon repair, is impaired [50]. The presence of neuronal‐intrinsic growth inhibitors within injured adult RGCs (summarized in Table 1) imposes an additional barrier to axon regeneration.

**TABLE 1:** List of putative neuronal‐intrinsic growth barriers that limit axon regeneration.

Gene candidate	Mechanism of growth‐inhibition	Pro‐regenerative effects after gene manipulations	References
PTEN	Inhibited mTOR‐mediated signaling pathways Impaired protein synthesis	Overexpression of microRNA‐19a suppressed PTEN expression in injured retinal ganglion cells (RGCs), leading to enhanced optic nerve regeneration. PTEN deletion promoted axon regeneration following optic nerve crush (ONC). Co‐deletion of PTEN and SOCS3 activated mTOR‐ and JAK/STAT‐mediated signaling pathways, sustaining long‐distance axon regeneration.	[38, 51, 52]
TSC1	Inhibited mTOR‐mediated signaling pathways, possibly via inactivation of Rheb	TSC1 deletion enhanced mTOR activity in injured RGCs, and facilitated robust axon regeneration after ONC.	[38]
SOCS3	Inhibited JAK/STAT‐mediated signaling pathways	SOCS3 deletion induced activation (phosphorylation) of STAT3 in injured RGCs, leading to robust optic nerve regeneration in a gp130‐dependent manner. CNTF acted synergistically to potentiate the pro‐regenerative effects induced by SOCS3 deletion to promote axon regeneration after ONC.	[52, 53]
PTPN2	Inhibited the activation of IFN‐γ/IFNGR/STAT1 axis	PTPN2 deletion potentiated the regenerative effects of IFN‐γ and sustained STAT1 activation, thereby promoting robust axon regeneration after ONC. Pharmaceutical inhibition of PTPN2 using novel inhibitor promoted optic nerve regeneration.	[54]
GSK3β	Negative regulator of axonal regrowth via inhibition of CRMP2 Negatively regulated the growth‐promoting effects induced by PTEN deletion via inhibition of mTOR activity in injured RGCs	GSK3β deletion promoted axon regeneration after ONC via disinhibition of CRMP2. GSK3β inhibition was crucial for the pro‐regenerative effects induced by PTEN deletion. Overexpression of CRPM2 alone was sufficient to overcome the growth inhibition induced by GSK3β and induced robust optic nerve regeneration.	[55, 56, 57]
KLF4	Putative transcriptional repressor that suppressed crucial genes for axonal outgrowth Inhibited STAT3 activity	KLF4 deletion induced robust axon regeneration in a STAT3‐dependent manner. Co‐deletion of KLF4 and SOCS3 amplified the pro‐regenerative effects induced by deletion of either gene alone.	[58, 59]
Kdm6a	Upstream regulator of KLF4 Inhibited genes crucial for axonal outgrowth	Kdm6a deletion stimulated robust regenerative growth following ONC, and potentiated the pro‐regenerative effects induced by PTEN deletion.	[60]
KLF9	Putative transcriptional repressor that suppressed crucial genes for axonal outgrowth Interacted with c‐Jun N‐terminal kinase 3 (JNK3) in RGCs	KLF9 deletion, or disruption of the interaction between KLF9 and JNK3, promoted optic nerve regeneration. KLF9 deletion and zinc chelation using TPEN sustained long‐distance axon regeneration following ONC.	[58, 61, 62]
MDM2/MDM4	Suppressed the expression of p53‐dependent regeneration‐associated genes (RAGs) Inactivated IGF1R‐mediated signaling (MDM4 only)	MDM4 deletion promoted the expression of p53‐dependent RAGs and enhanced axon regeneration after ONC. Pharmaceutical inhibition of MDM2 using Nutlin‐3a up‐regulated the expression of p53, promoted the expression of p53‐dependent RAGs, and enhanced axon regeneration after ONC.	[63]
REST and CTCF	Potent transcriptional repressors of key pro‐regenerative signaling pathways associated with axon regeneration in the adult CNS	REST and CTCF deletion independently enhanced the expression of many RAGs, enabling robust axon regeneration after ONC.	[64, 65]
Synaptic proteins (e.g., Cacna2d2, RIM1/2, and Munc13)	Suppressed the neuronal‐intrinsic regenerative capacity via unknown mechanisms	Deletion of these synaptic proteins alone promoted axon regeneration following peripheral nerve injury and spinal cord injury (SCI). Overexpression of enhancer of zeste homolog 2 (Ezh2) suppressed the expression of genes related to synaptic functions, leading to enhanced optic nerve regeneration.	[66, 67, 68]
Myosin IIA/B	Negative regulators of growth cone formation that initiate growth cone collapse into retraction bulbs at the distalmost tips of regenerating axons	Knockout of myosin IIA/B in injured RGCs sustained long‐distance axon regeneration following ONC.	[69]
Fmn2	Negative regulators of microtubule dynamics via HDAC5‐dependent mechanisms	Gene silencing of Porf‐2 promoted axon regeneration and functional recovery following peripheral nerve injury in mice.	[33]
Porf‐2	Negative regulator of growth cone formation that constrains growth cone expansion and initiate growth cone collapse via EphB‐mediated signaling pathways	Gene silencing of Porf‐2 promoted axon regeneration and restored pupillary light reflex (PLR) following ONC. The pro‐regenerative effects of Porf‐2 silencing were mediated by increased Rac1 activity, which in turn stimulates growth cone formation at the distal tips of regenerating RGC axons.	[70, 71]
Syntaphilin	Negative regulator of axonal mitochondrial transport	Deletion of syntaphilin facilitated effective axonal mitochondrial transport, thereby promoting axon regeneration after peripheral nerve injury and SCI.	[72, 73]
Lipin1 and DGAT1/2	Control the synthesis of glycerolipid for energy storage	Deletion of Lipin1 or DGAT1/2 redirected lipid metabolism from triglyceride synthesis to the production of membrane‐forming phospholipid, and thereby facilitated robust axon regeneration following ONC. Deletion of Lipin1 increased phosphatidic acid levels, leading to activation of mTOR‐ and JAK/STAT‐mediated signaling pathways for regenerative growth in the adult CNS.	[74, 75]

### 3.1 PTEN and SOCS3: Two Roadblocks to mTOR and JAK/STAT Signaling Pathways Essential for CNS Regeneration

**FIGURE 2:**
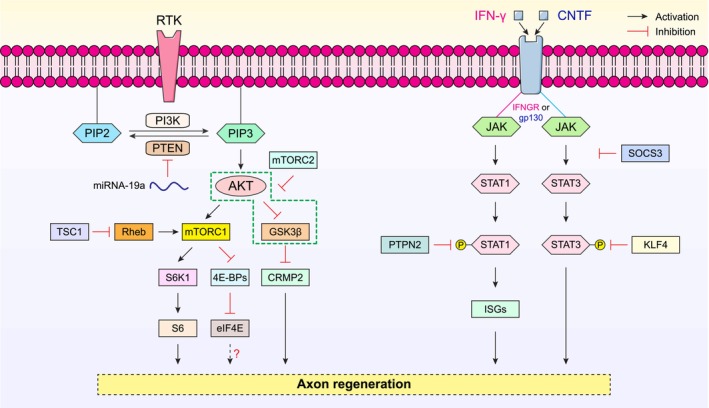
Simplified schematic diagram illustrating the roles of mTOR and JAK/STAT signaling pathways in the regulation of axon regeneration. Left panel: PTEN is a key negative regulator of phosphatidylinositol 3‐kinase (PI3K)‐mediated signaling, dephosphorylating PIP3 to PIP2. PTEN deletion leads to the activation of AKT and its downstream effector mTORC1, resulting in increased phosphorylation of ribosomal protein S6 (a S6K1 substrate) and enhancing axon regeneration after optic nerve crush (ONC). Concurrently, mTORC1 inhibits the eukaryotic translation initiation factor 4E‐binding protein (4E‐BP), releasing eIF4E to assemble into the eIF4F complex for cap‐dependent translation initiation. PTEN deletion‐induced AKT activation suppresses GSK3β activity, causing CRMP2 disinhibition that promotes axon regeneration after ONC. Notably, mTORC2 inhibits AKT‐mediated suppression of GSK3β and negatively regulates axon regeneration. TSC1 and Rheb function as upstream regulators of mTORC1: TSC1 deletion or Rheb overexpression enhances mTOR activity; both of which induce robust axon regeneration following ONC. Right panel: JAK/STAT‐mediated axon regeneration involves two primary receptor systems: the interferon‐gamma receptor (IFNGR) and the gp130 cytokine receptor. This pathway is activated by ligands such as interferon‐gamma (IFN‐γ) and ciliary neurotrophic factor (CNTF), which signal through the binding to IFNGR and gp130, respectively. SOCS3 and KLF4 are potent negative regulators of the JAK/STAT signaling by suppressing STAT3 phosphorylation. Deletion of either KLF4 or SOCS3 alone sufficiently drives substantial axon regeneration following ONC. Additionally, PTPN2 deletion amplifies IFN‐γ response and induces STAT1 phosphorylation in injured RGCs, stimulating the expression of interferon‐stimulated genes (ISGs) to promote axon regeneration.

**FIGURE 3:**
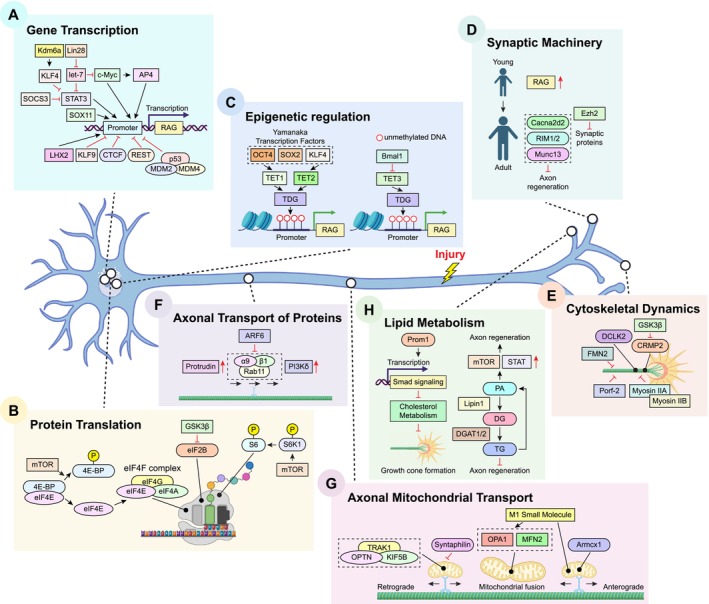
Neuronal‐intrinsic mechanisms driving effective axon regeneration following nerve injury. (A) Multiple transcription factors, including STAT3, c‐Myc (and its downstream target AP4), SOX11, and LHX2, control the (re‐)expression of intrinsic transcriptional programmes required for effective axonal outgrowth (i.e., regeneration‐associated genes; RAGs) following axotomy. Lin28 acts post‐transcriptionally to suppress let‐7 microRNA maturation, thereby enabling expression of key RAGs such as c‐Myc and STAT3 to initiate intrinsic regenerative programmes. Conversely, several transcriptional repressors, such as KLF4 (which antagonizes STAT3 activity), Kdm6a, KLF9, SOCS3 (which inhibits STAT3 phosphorylation), REST, CTCF, and the MDM2/MDM4 complex (which blocks the p53‐dependent transcriptional pathway), restrict the reactivation of RAGs, thereby limiting axonal regrowth in the adult mammalian CNS. (B) mTOR serves as a critical regulator of protein translation through its effectors S6K1 and 4E‐BP. Phosphorylation of S6K1 enhances translation through ribosomal protein S6, whereas phosphorylation of 4E‐BP releases its binding with eIF4E, enabling assembly of the eIF4F complex to initiate cap‐dependent protein translation—both processes contribute to enhanced axonal regeneration. In contrast, GSK3β inhibits eIF2B‐dependent protein synthesis, thereby suppressing axonal regrowth. (C) In the adult PNS, up‐regulation of RAGs following axotomy is driven by TET3‐mediated DNA demethylation. Conversely, the core circadian clock transcriptional regulator Bmal1 suppresses TET3 expression, thereby restricting TET3‐mediated DNA demethylation and up‐regulation of RAGs. Additionally, three Yamanaka transcription factors (OCT4, SOX2, and KLF4) restore DNA methylation patterns observed in developing neurons to drive RAG expression via TET1‐ and TET2‐dependent mechanisms, thereby promoting CNS regeneration. (D) Components of the synaptic vesicle machinery, including Cacna2d2, RIM1/2, and Munc13, have emerged as neuronal‐intrinsic growth inhibitors of axon regeneration. Deletion of these genes individually promotes robust axonal regrowth in the adult CNS. (E) Deletion of GSK3β promotes disinhibition of CRMP2, which in turn enhances microtubule dynamics and growth cone formation at the distal tips of regenerating axons, leading to enhanced axon regeneration. Gene silencing of Fmn2 also promotes axonal regrowth by augmenting microtubule dynamics at the distal axons. Similarly, DCLK2 overexpression facilitates growth cone formation, leading to improved optic nerve regeneration. Co‐deletion of non‐muscle myosin IIA/B, or gene silencing of Porf‐2, effectively remodels growth cone morphology at distal regenerating RGC axons and enhances the efficiency of RGC axonal pathfinding, resulting in improved optic nerve regeneration. (F) In the adult optic nerve, axonal transport of pro‐regenerative α9β1 integrin is largely restricted. However, inactivation of ARF6 or activation of PI3Kδ enables effective delivery of active α9β1 integrin to the growth cone. Similarly, protrudin overexpression drives axonal transport of Rab11 vesicles and α9β1 integrin, leading to enhanced axon regeneration following ONC. (G) Promoting axonal mitochondrial transport has emerged as a therapeutic target for enhancing regenerative growth. Deletion of the mitochondria‐anchoring protein syntaphilin, or overexpression of Armcx1 or the OPTN/KIF5B/TRAK1 complex, induces robust axon regeneration following CNS injury. Similarly, the mitochondrial fusion promoter M1 small molecule stimulates long‐distance optic nerve regeneration via OPA1‐ and MFN2‐dependent mechanisms. (H) Altered lipid metabolism has recently been implicated in regeneration failure in the adult CNS. Redirecting lipid biosynthesis from triglyceride (TG) synthesis for storage towards phospholipid synthesis (such as phosphatidic acid; PA) via deletion of Lipin1 and DGAT1/2 induces robust regeneration following ONC, possibly through increased activation of mTOR and JAK/STAT signaling pathways. Prom1 overexpression reduces cellular cholesterol levels via Smad‐mediated signaling pathways. Cholesterol depletion is known to promote the enlargement of growth cone and facilitate axonal outgrowth.

The mammalian target of rapamycin (mTOR) signaling pathway (Figure 2), a key modulator of cellular metabolism, controls many crucial cellular processes such as protein synthesis, cell growth, proliferation, survival, migration, and cytoskeleton remodeling [76]—all of which are essential for successful axon regeneration [77]. In developing RGCs, mTOR activity is maintained at a high level, which supports robust axon elongation. However, its activity declines sharply upon maturation as RGC axons reach their target regions in the brain and is further suppressed in injured RGCs following axonal injury [38]. This marked reduction in mTOR activity is, at least in part, due to a significant decrease in microRNA‐19a (Figure 2)—a recently identified upstream regulator of phosphatase and tensin homolog (PTEN) expression during development. The down‐regulation of microRNA‐19a results in a gradual increase in PTEN levels as RGCs mature [51]. Neuronal‐specific deletion of PTEN or tuberous sclerosis complex 1 (TSC1), both of which negatively regulate the mTOR pathways (Figure 2), restored mTOR activity in injured RGCs, which promoted significant axon regeneration and enhanced RGC survival following ONC in mice [38]. Similarly, activation of STAT3‐mediated signaling (Figure 3A) played a critical role in the injury‐induced transcriptomic responses for the enhanced regenerative capacity in pre‐conditioned peripheral neurons [78]. In contrast, optic nerve injury failed to induce STAT3 activation in damaged RGCs, which contributed to regeneration failure in the adult mammalian CNS [52]. Notably, in the mouse ONC model, the deletion of suppressor of cytokine signaling 3 (SOCS3), a negative regulator of the JAK/STAT signaling, facilitates robust axon regeneration in a gp130‐dependent manner (Figure 2). The pro‐regenerative effects of SOCS3 deletion are further enhanced by exogenous administration of ciliary neurotrophic factor (CNTF), a gp130 receptor ligand [53]. Additionally, co‐deletion of PTEN and SOCS3 activated both mTOR‐ and JAK/STAT‐mediated signaling pathways, which acted synergistically to sustain long‐distance axon regeneration in ONC mice [52]. This co‐activation enabled reinnervation of the hypothalamic suprachiasmatic nucleus [79] in which the regenerating axons further extended caudally into the optic tracts [52]. More strikingly, deletion of another intrinsic growth repressor, protein tyrosine phosphatase non‐receptor type 2 (PTPN2) (Figure 2), in injured RGCs, coupled with a low dose of interferon‐gamma (IFN‐γ), remarkably amplified the already robust regenerative responses induced by PTEN/SOCS3 co‐deletion. This synergistic intervention propelled regenerating RGC axons to traverse the entire length of the optic nerve and reach the optic chiasm within two weeks following ONC [54].

### 3.2 PI3K/AKT/mTOR Signaling and Its Interplay With GSK3β in CNS Regeneration

The PI3K/AKT/mTOR signaling pathway plays a central role in promoting regenerative growth in the adult mammalian CNS [38, 80, 81] (Figure 2). Deletion of the intrinsic growth repressor PTEN activates this signaling cascade, driving robust optic nerve regeneration via downstream effectors such as mTOR, a serine/threonine protein kinase that forms the mTORC1 complex. This complex regulates essential cellular processes, including cell growth, motility, and survival via the activation of PI3K/AKT pathway [82] (Figure 2). mTORC1 also regulates the activity of eukaryotic translation initiation factors, 4E‐binding protein (4E‐BP), and ribosomal S6 kinases (S6Ks) (Figure 3B). Phosphorylation of 4E‐BP by mTORC1 releases its inhibitory binding to eIF4E (Figure 2), enabling the assembly of the eIF4F complex for cap‐dependent translation initiation [83] (Figure 3B). Similarly, mTOR activation induces phosphorylation of S6Ks to initiate protein synthesis [84] (Figures 2 and 3B). Interestingly, manipulating mTORC1 effectors reveals intriguing dynamics in axon regeneration: overexpression of a constitutively active form of S6K1, but not deletion of the translational repressor 4E‐BP, could partially recapitulate the pro‐regenerative effects of PTEN deletion in ONC mice. However, combining S6K1 overexpression with PTEN deletion paradoxically diminished the regenerative growth observed with PTEN deletion alone. This reduced outgrowth was likely attributed to a negative feedback loop within the insulin receptor/PI3K signaling pathway, driven by enhanced S6K1 activity, which phosphorylated insulin receptor substrate 1 (IRS‐1) and ultimately suppressed AKT activity in injured RGCs [85]. Among the three AKT isoforms, AKT1 and AKT3 were predominantly expressed in adult RGCs, whereas AKT2 was expressed at much lower levels. Overexpression of AKT3 induced more robust axon regeneration than AKT1 following ONC. Notably, AKT phosphorylation at distinct sites exerted contrasting effects on optic nerve regeneration: phosphorylation at T308 promoted axon regrowth via mTORC1 activation and GSK3β inhibition (Figure 2), whereas phosphorylation at S473 was growth‐inhibitory due to mTORC2‐mediated suppression of GSK3β inactivation [55]. GSK3β itself was identified as a potent intrinsic growth inhibitor (Figure 2). Its activation antagonizes the pro‐regenerative effects of AKT, whereas GSK3β deletion alone was sufficient to promote a considerable extent of axon regeneration after ONC [55, 86] through an mTORC1‐independent but eIF2B‐dependent mechanism by reducing the extent of eIF2Bε phosphorylation (i.e., elevated eIF2Bε activity) to promote global protein synthesis (Figure 3B) and support regenerative growth [86]. Furthermore, GSK3β inactivation induced by PTEN deletion led to the disinhibition of collapsin response mediator protein 2 (CRMP2), a key effector for axonal growth (Figure 2). Restoring CRMP2 activity has been shown to overcome the growth‐inhibitory effects of constitutively active GSK3β [56, 57, 87]. Additionally, overexpression of an active form of CRMP2 alone was sufficient to drive regenerative growth of RGC axons following ONC [88]. The GSK3β/CRMP2 axis is also known to regulate microtubule dynamics, a key factor in the formation of the growth cone required for regenerative growth [87, 89, 90, 91], further emphasizing the importance of this signaling cascade in regulating axon regeneration.

In contrast to PTEN and TSC1 [38], ras homolog enriched in brain (Rheb) was identified as a positive regulator of mTOR signaling to promote optic nerve regeneration (Figure 2) [92, 93]. The pro‐regenerative effects of Rheb were mediated by mTORC1 downstream effectors, requiring both S6K1 activation and 4E‐BP inhibition [93]. Collectively, these studies underscored the complexity and interdependence of the PI3K/AKT/mTOR pathway and associated signaling networks in regulating axon regeneration. Of note, while AKT activation was crucial for PTEN deletion‐induced optic nerve regeneration, PTEN deletion alone only induced modest AKT activation. The combination of PTEN deletion with either AKT overexpression or GSK3β deletion synergistically enhanced the extent of axonal regrowth in ONC mice compared to PTEN deletion alone [94]. This indicated that PTEN deletion might trigger both AKT‐dependent and AKT‐independent mechanisms to drive effective nerve repair [94]. Despite nearly two decades of research documenting the pro‐regenerative effects of PTEN deletion, the precise underlying mechanisms remained incompletely understood.

### 3.3 Transcriptional Repressors That Restrict Neuronal Regenerative Programmes

In an in vitro screening of axonal outgrowth suppressors, the zinc‐finger transcription factor Krüppel‐like factor 4 (KLF4) was identified as a potent growth inhibitor that restricted neurite outgrowth in cultured RGCs. Genetic ablation of KLF4 in injured RGCs promoted axon regeneration following ONC [58]. Additionally, KLF4 interacted with STAT3 and suppressed its activity by binding to its phosphorylated form (Figure 3A). Deleting KLF4 released its inhibition on STAT3 activation, thereby further enhancing regenerative growth in these injured RGCs. Remarkably, co‐deletion of KLF4 and SOCS3 produced a synergistic effect, further amplifying axonal regrowth in ONC mice compared with deletion of either gene alone [59]. Recently, Kdm6a was identified as an upstream regulator of KLF4: deletion of Kdm6a substantially down‐regulated KLF4 expression in injured RGCs through epigenetic mechanisms, thereby enhancing axon regeneration following ONC (Figure 3A). Conversely, overexpressing KLF4 completely abolished the pro‐regenerative effects induced by Kdm6a deletion [60]. Similarly, another KLF family member KLF9 (Figure 3A) also acted as a potent growth suppressor to impede regrowth of RGC axons following axotomy [58]. Silencing this gene alone [61], or in combination with zinc chelation using TPEN [62], has been shown to significantly enhance optic nerve regeneration following ONC. Remarkably, some regenerating axons in KLF9‐knockdown + TPEN‐treated mice extended beyond the optic chiasm and reinnervated the hypothalamic suprachiasmatic nucleus six weeks post‐ONC [62]. A protein–protein interaction network analysis identified a protein complex comprising MDM4, MDM2, and p53 as a potent inhibitor of axonal regrowth in injured RGCs (Figure 3A). Deletion of MDM4 enhanced the activation of p53‐dependent RAGs, including p21, Gap43, Cap23, and Stmn2 (also known as SCG10) [63], all of which are crucial for successful axon regeneration [77]. Deletion of this gene also promoted regenerative growth of RGC axons via the activation of insulin‐like growth factor 1 receptor (IGF1R)‐mediated signaling pathways following ONC [63]. Similarly, pharmaceutical inhibition of MDM2 using Nutlin‐3a enhanced axon regeneration following ONC, achieving pro‐regenerative effects comparable to MDM4 deletion via activation of p53‐dependent RAGs [63].

More recently, a transcription network analysis has predicted two transcriptional repressors, RE1‐Silencing Transcription Factor (REST) and CCCC‐binding factor (CTCF) as potential upstream repressors that inhibit the activation of pro‐regenerative transcriptomic programmes in the adult CNS. Sustained elevated REST expression in injured CNS neurons suppressed the activation of key signaling pathways for axon regeneration (Figure 3A), including cAMP‐mediated, Ephrin‐, PKA‐, TGFβ‐, GPCR‐, MAPK‐, and integrin signaling pathways. Notably, genetic ablation of REST or CTCF alone in injured RGCs resulted in significant axon regeneration following ONC, supporting their role as negative regulators of axon regeneration [64, 65]. However, contrasting findings have emerged from studies of peripheral nerve injury. REST expression was transiently elevated in response to peripheral nerve injury but returned to baseline levels due to the up‐regulation of its upstream repressor, ubiquitin‐like containing PHD ring finger 1 (UHRF1) at later time points. Interestingly, pharmacological inhibition of REST impaired axonal outgrowth in adult DRG neurons [95]. In another study, CTCF, alongside another chromatin remodeling factor cohesin, has been implicated as key facilitators of regenerative phenotypes in injured peripheral neurons. Deletion of either gene is sufficient to impair peripheral nerve regeneration following axotomy [96, 97]. Similarly, in response to peripheral nerve injury, the histone acetyltransferase p300/CBP‐associated factor (PCAF) promoted histone acetylation, thereby activating numerous RAGs in injured peripheral neurons. Overexpression of PCAF alone was sufficient to drive robust regeneration of sensory axonal fibers across the lesion site following spinal cord injury (SCI) [98]. These apparent discrepancies in the roles of REST, CTCF, and other transcriptional regulators may reflect fundamental differences in the transcriptomic programmes required for nerve regeneration in the CNS and PNS. Alternatively, it might also indicate that distinct sets of genes are required at different phases of axonal outgrowth, whose regulation varies with neuronal context [64, 95].

### 3.4 Epigenetic Control of Intrinsic Regenerative Programmes

In injured PNS neurons, epigenetic regulators methylcytosine dioxygenases (TETs) play a crucial role in mounting regenerative phenotypes following axotomy. TET3 and its downstream mediator thymine DNA glycosylase (TDG) facilitate DNA demethylation, leading to the re‐expression of key RAGs to facilitate regenerative growth in the injured PNS neurons (Figure 3C), while TET1 is required for regenerative responses induced by PTEN deletion following ONC [99]. Interestingly, the circadian clock regulator Bmal1 appears to function as an upstream repressor of TET3, limiting its DNA demethylation activity and subsequent reactivation of RAGs in injured PNS neurons (Figure 3C). Conditional deletion of Bmal1 enhanced axon regeneration after peripheral nerve injury [100]. However, another finding suggests that Bmal1 expression is essential for the regenerative growth of peripheral axons in some contexts [101]. These discrepancies highlight the complexity of circadian clock‐mediated epigenetic modulation and raise important questions about how Bmal1‐dependent regulation of TET3 influences axonal regeneration. Further investigation is required to elucidate the interplay between circadian rhythms and DNA demethylation in controlling the regenerative capacity of injured neurons, particularly in the non‐regenerative CNS neurons such as RGCs.

### 3.5 Synaptic Machinery as Putative Negative Regulators of Axon Regeneration

During neuronal maturation, a transition from a growth‐competent state to an electrically active state occurs as neurons start innervating their targets. This process is characterized by the up‐regulation of genes involved in synapse formation, which might paradoxically suppress their neuronal‐intrinsic regenerative capacity [102]. Emerging evidence suggests that synaptic proteins such as Cacna2d2 [encoding the α2δ2 subunit of voltage‐gated calcium channels (VGCCs) critical for vesicle release], Rab3‐interacting molecule 1/2 (RIM1/2), and Munc13 act as intrinsic growth inhibitors, limiting neurite outgrowth in the highly regenerative cultured DRG neurons (Figure 3D). To counteract this growth inhibition, pharmacological interventions targeting these synaptic proteins have shown promise. For example, the VGCC inhibitor pregabalin suppresses Cacna2d2‐mediated signaling, while baclofen inhibits Munc13‐dependent VGCC activation. Both approaches attenuate synaptic transmission and enhance in vivo axon regeneration after SCI [66, 67]. Similarly, baclofen‐mediated suppression of aberrant cerebellar hyperexcitability has been shown to correct neurotransmitter imbalance in the brain, thereby accelerating motor functional recovery in mouse models of ataxia following severe peripheral nerve injury [103], consistent with previous studies demonstrating that an imbalance between excitatory and inhibitory neurotransmitters contributed to the impaired functional recovery following peripheral nerve injury [104, 105]. Nonetheless, the contribution of these synaptic proteins to growth inhibition in injured RGCs remains to be determined in the mouse ONC model.

A recent study identified the histone methyltransferase, enhancer of zeste homolog 2 (Ezh2), as a key epigenetic regulator controlling the expression of genes associated with ion transport and synaptic transmission (Figure 3D). Consistent with previous findings, overexpression of Ezh2 in injured RGCs suppressed the expression of genes related to synaptic functions, notably Slc6a13, which encodes the GABA transporter 2 (GAT2), thereby enhancing axon regeneration following ONC [68]. Together, these findings support the emerging concept that reverting injured neurons from an electrically active state to an electrically silent, growth‐competent state can restore their intrinsic regenerative capacity, including that of RGCs [106].

### 3.6 Other Negative Regulators of Axon Regeneration Identified by Genome‐Wide Screens

Recent advances in high‐throughput loss‐of‐function screening have enabled the functional evaluation of thousands of protein‐coding genes, both in vitro and in vivo, for their roles in regulating axonal outgrowth. A short hairpin RNA (shRNA)‐mediated gene silencing screen identified 479 putative growth‐inhibitory genes, where suppression of individual gene candidates enhanced neurite outgrowth in cultured mouse cortical neurons. Notably, in vivo silencing of genes implicated in PI3K/AKT signaling modulation (Innpl1), cytokine signaling (Socs4 and Il‐22), negative regulation of IGF‐mediated signaling (Airn), or intracellular organelle transport (Rab27b) significantly promoted axon regeneration following ONC [107, 108]. Among these, Rab27b has been linked to mediating the anterograde transport of tropomyosin receptor kinase B (TrkB) [109], a receptor recently shown to induce robust axon regeneration after ONC [110]. In that case, Rab27b appeared to exert its inhibitory effects via mechanisms independent of TrkB transport. Instead, its potential growth‐inhibitory effects are likely associated with its role in regulating synaptic vesicle release [111], a hypothesis that warrants further investigation.

More recently, an in vivo CRISPR/Cas9‐mediated genome‐wide screen was performed to identify repressors of axon regeneration using the mouse ONC model [65]. Of the 1893 transcription factors screened, 13 were identified as negative regulators of optic nerve regeneration, including genes involved in epigenetic regulation (CTCF, EP300, Sin3a, and Rbbp7), basic helix‐loop‐helix transcription factors (Tcf3 and Tcf24), LIM‐homeobox transcription factors (Lhx2 and Lhx6), and other transcriptional regulators (Pawr, Carf, Stag1, and Ebf3). Deletion of these 13 genes individually resulted in robust axon regeneration following ONC [65]. Interestingly, conflicting results have been reported regarding Lhx2, with a subsequent study demonstrating that Lhx2 overexpression promoted long‐distance axon regeneration after ONC [112]. In adult retinae, Lhx2 is predominantly expressed in Müller glia and amacrine cells (inhibitory interneurons that innervate RGCs), with minimal expression detected in RGCs themselves [113]. Critically, AAV2‐mediated gene delivery driven by universal promoters transduces both RGCs and small subsets of amacrine cells [36, 114]. While AAV2‐mediated Lhx2 deletion in the first study was driven by a universal promoter (CAG) [65], the observed pro‐regenerative effects may have been cell non‐autonomous, potentially arising from Lhx2 deletion in amacrine cells rather than RGCs. In contrast, the second study employed RGC‐specific Lhx2 overexpression, which resulted in long‐distance optic nerve regeneration [112], suggesting a cell‐autonomous pro‐regenerative effect within RGCs. These findings raise an intriguing question: could simultaneous deletion of Lhx2 in amacrine cells combined with Lhx2 overexpression in RGCs elicit even more robust axon regeneration following ONC? Given that ONC aberrantly elevates the activity of amacrine cells, which suppresses growth factor signaling and thereby represses the regenerative potential of RGCs [115], such experiments could elucidate how Lhx2 modulates the interplay between RGCs and amacrine cells to regulate axon regeneration.

## 4 Restoring the Expression of Genes Crucial for Axonal Regrowth to Enhance Optic Nerve Regeneration

In addition to the presence of intrinsic growth suppressors, inactivation of genes involved in transcriptional regulation, cellular growth, metabolism, integrin signaling, growth cone formation, and mitochondrial axonal transport, as well as altered lipid metabolism in injured CNS neurons represents additional barriers that limit axon regeneration (summarized in Table 2). Targeting these pathways to restore the intrinsic regenerative capacity of injured RGCs holds considerable promise for promoting nerve repair.

**TABLE 2:** List of crucial regulators that enable robust axon regeneration following axotomy.

Gene candidate	Mechanism of growth‐stimulation	Pro‐regenerative effects after gene manipulations	References
c‐Myc	Putative master transcription factor to regulate genes involved in anabolic metabolism Enhanced mTOR activity in injured RGCs Modulated the expression of regeneration‐associated genes (RAGs)	Overexpression of c‐Myc promoted optic nerve regeneration to an extent comparable to PTEN deletion. Overexpression of c‐Myc further potentiated the pro‐regenerative effects induced by PTEN and SOCS3 co‐deletion.	[116, 117]
AP4	Downstream effector of c‐Myc Enhanced mTOR activity in injured RGCs	Overexpression of AP4 induced robust axon regeneration following optic nerve crush (ONC). AP4 expression was crucial for the pro‐regenerative effects induced by c‐Myc overexpression and PTEN deletion.	[114]
DCLK2	Initiated growth cone formation following axotomy	Overexpression of DCLK2 promoted optic nerve regeneration and potentiated the pro‐regenerative effects of PTEN deletion. DCLK2 was required for regenerative growth induced by PTEN deletion.	[118]
SOX11	Reactivated the axon‐growth programme utilized in developmental stages	Overexpression of SOX11 promoted axon regeneration in non‐α‐RGCs but selectively eliminated α‐RGCs.	[119]
Lin28	Activated AKT/mTOR‐mediated signaling pathways in injured RGCs Suppressed hyperactivity of amacrine cells and restored the RGC responsiveness to IGF‐1	Overexpression of Lin28 induced robust axon regeneration following ONC. Overexpression of Lin28 potentiated the pro‐regenerative effects induced by PTEN deletion. Lin28 overexpression in amacrine cells promoted regeneration of RGC axons, whilst Lin28 overexpression in RGCs alone failed to elicit regenerative responses following ONC.	[115, 120, 121]
Kindlin‐1/α9	Activated integrin signaling for robust axonal outgrowth Effective axonal transport of α9β1 integrin towards growth cones of the regenerating axons	Overexpression of kindlin‐1 and α9 supported long‐distance axon regeneration after spinal cord injury (SCI) via enhanced axonal transport and trafficking along regenerating axons.	[122, 123, 124, 125]
Protrudin	Facilitated effective axonal transport of Rab11 vesicles and α9β1 integrin	Overexpression of protrudin enhanced axonal transport of growth machinery and supported long‐distance axon regeneration following ONC.	[126]
Armcx1	Facilitated axonal mitochondrial transport in the regenerating axons	Overexpression of Armcx1 promoted axon regeneration following ONC. Armcx1 expression was crucial for the regenerative effects induced by PTEN and SOCS3 co‐deletion.	[127]
Prom1	Negative regulator of cholesterol metabolism via Smad‐mediated signaling pathways	Overexpression of Prom1 promoted axon regeneration after peripheral nerve injury.	[128]
Lhx2	Promoted the transcription of RAGs Inhibited the transcription of semaphorin 3C (Sema3C)	Overexpression of Lhx2 induced robust axon regeneration following ONC, and synergised the pro‐regenerative effects of CNTF. Overexpression of Lhx2 preserved visual function in a NMDA‐induced excitotoxicity model. Overexpression of Lhx2 promoted RGC survival in a microbead‐induced ocular hypertension glaucoma model.	[112]
F‐iTrkB	Sustained activation BDNF–TrkB signaling pathways	Overexpression of F‐iTrkB protected the GLAST‐KO mice (a mouse model for NTG) from visual functional decline. Overexpression of F‐iTrkB induced long‐distance axon regeneration following ONC.	[110]
Yamanaka Transcription Factors (OCT4/SOX2/KLF4)	Restore youthful DNA methylation patterns and transcriptomes via TET1‐ and TET2‐dependent DNA demethylation mechanisms	Overexpression of these three Yamanaka transcription factors induced robust axon regeneration following ONC. Overexpression of these three Yamanaka transcription factors restored visual functions in a microbead‐induced ocular hypertension glaucoma model.	[129]
OPTN	Facilitated robust axonal mitochondrial transport by anchoring the KIF5B‐TRAK1‐mitochondria complex to axonal microtubules	Overexpression of OPTN, alongside KIF5B and TRAK1, promoted axon regeneration following ONC. Overexpression of OPTN/KIF5B/TRAK1 prevented visual functional deficits in SOHU glaucoma mice.	[130]

### 4.1 Transcriptional and Post‐Transcriptional Regulation of Intrinsic Regenerative Programmes

In response to ONC, the expression of c‐Myc (a master regulator of cellular metabolism) declined sharply in injured RGCs. Restoring c‐Myc expression in the injured neurons stimulated robust axon regeneration following ONC [116], potentially by modulating the RAG expression following axotomy [117] (Figure 3A). Remarkably, its pro‐regenerative effects were markedly amplified with the co‐deletion of PTEN and SOCS3 as many regenerating axonal fibers crossed the optic chiasm and extended into the optic tracts in the brain observed in ONC mice [116]. A recent study demonstrated that c‐Myc‐induced optic nerve regeneration was dependent on the expression of its downstream effector, AP4 (Figure 3A). Notably, following ONC, AP4 alone proved sufficient to drive substantial axon regeneration, even in the absence of c‐Myc [114]. More importantly, the pro‐regenerative responses induced by PTEN deletion also relied on this c‐Myc‐AP4 axis: silencing either c‐Myc or AP4, or both, eliminated the axonal regrowth normally seen in PTEN‐deleted ONC mice [114, 116].

The transcription factor SOX11 (Figure 3A), crucial for cell‐fate specification during development, was markedly down‐regulated in mature RGCs. Overexpression of SOX11 promoted robust axon regeneration by up‐regulating genes involved in axon growth and down‐regulating those associated with synaptic transmission [119]. This transcriptional shift supports the emerging concept that successful axon regeneration requires reverting adult RGCs from an electrically active state back to a growth‐competent state [131, 132]. However, this pro‐regenerative effect occurred exclusively in non‐α‐RGCs. Paradoxically, it caused a selective loss of α‐RGCs following ONC [119]. By contrast, PTEN deletion supported axon regeneration in α‐RGCs only [133]. Combining both manipulations (i.e., PTEN deletion with SOX11 overexpression) enhanced the pro‐regenerative effects of PTEN deletion alone, enabling long‐distance axon regeneration from non‐α‐RGCs, although most of the α‐RGCs were still eliminated due to SOX11 overexpression [119].

STAT3 is a crucial transcription factor that drives intrinsic regenerative responses following peripheral nerve lesion [78]. While SOCS3 deletion induced phosphorylation (activation) of STAT3, enabling robust axon regeneration after ONC [52, 53], overexpression of a constitutively active variant of STAT3 induced only modest axon regeneration [134]. However, overexpression of a “hyperactivated” STAT3 variant, generated by fusing the VP16 transactivation domain with constitutively active STAT3, promoted robust axon regeneration following ONC [134] to an extent similar to those observed with SOCS3 deletion [53]. These pro‐regenerative effects are, at least in part, mediated by enhanced transcriptional activity and the up‐regulation of STAT3‐dependent RAGs (Figure 3A), including Atf3 and Sprr1a [134].

RNA‐binding protein Lin28, a regulator of cellular development and differentiation [135], is typically undetectable in mature cells [136]. In line with this, Lin28 expression declined sharply in adult CNS neurons, including RGCs and corticospinal neurons, upon maturation [120]. However, Lin28 was re‐expressed in the injured PNS neurons following axotomy to peripheral nerves, and this up‐regulation was critical for regenerative growth in the adult PNS [121]. The pro‐regenerative effects of Lin28 were partly mediated through its regulation of the post‐transcriptional processing of specific microRNA (miRNA) [121], most notably pre‐let‐7, by blocking its maturation into the functional miRNA [137]. Since mature let‐7 miRNAs suppress the expression of key RAGs such as c‐Myc and Stat3 [138, 139] (Figure 3A), Lin28 up‐regulation may help maintain a pro‐regenerative intrinsic growth programme that supports robust axon regeneration in the PNS [121]. By contrast, ONC failed to induce Lin28 up‐regulation in injured RGCs [120]. Nonetheless, overexpression of Lin28a restored the regenerative potential of RGCs by enhancing mTOR signaling within retinal neurons, leading to robust axon regeneration after ONC [120, 121], and acted synergistically with PTEN silencing to further enhance regenerative growth [121]. Interestingly, the robust regenerative response induced by Lin28 overexpression appeared to be non‐neuronal‐intrinsic: while overexpression of Lin28 in amacrine cells suppressed their hyperactivity in response to ONC and reinstated RGC responsiveness to growth factors (i.e., IGF‐1), thereby enhancing optic nerve regeneration, overexpression in RGCs alone failed to reproduce such regenerative phenotypes in ONC mice [115].

### 4.2 Modulating Cytoskeletal Dynamics to Drive Robust Axonal Growth

Effective axon regeneration relies on precise cytoskeletal remodeling [106], yet ONC suppresses the expression of doublecortin‐like kinase 1 and 2 (DCLK1/2) [118], which are essential for growth cone formation [140] (Figure 3E). Restoration of DCLK2 expression significantly enhanced the formation of growth‐competent growth cones in retinal explants and supported lengthy regrowth of RGC axons in vivo [118]. Similarly, gene silencing of Porf‐2, or genetic ablation of non‐muscle myosin IIA and IIB, significantly enhanced axon regeneration following ONC by modulating cytoskeletal dynamics (Figure 3E) and thereby promoting the formation of growth‐competent growth cones [69, 70]. The pro‐regenerative effects of Porf‐2 silencing were likely mediated through modulation of EphB1/Rac1‐dependent cytoskeletal remodeling [71]. More strikingly, knockdown of Porf‐2 in injured RGCs not only preserved their neuronal viability but also maintained their functional integrity, as evidenced by the restoration of electroretinogram (ERG) activity and pupillary light reflex (PLR) [70], a key indicator of re‐established functional connectivity between RGCs and the brainstem, enabling proper pupil constriction in response to light [36, 37]. Interestingly, the regenerative benefits of myosin II inhibition extended beyond CNS injury, as local administration of a novel non‐muscle myosin II inhibitor (a blebbistatin analogue) at the injury site promoted axonal regrowth and facilitated early motor functional recovery following peripheral nerve injury in mice [141]. Similarly, we recently identified FMN2 as a negative regulator of microtubule dynamics. Silencing of this gene accelerated in vivo axon regeneration and improved functional recovery following sciatic nerve injury (Figure 3E), although its pro‐regenerative effects in the CNS remained to be determined [33].

### 4.3 Integrin Signaling and Axonal Transport in CNS Regeneration

The composition of the extracellular matrix (ECM) surrounding the lesion core and penumbra undergoes significant changes following CNS injuries such as SCI and ONC [142]. Certain ECM components, such as chondroitin sulfate proteoglycans (CSPGs), are potent barriers to axon regeneration, contributing substantially to the growth‐restrictive microenvironment that hampers neural repair [19]. Therapeutic interventions targeting CSPG degradation have demonstrated considerable promise in enhancing functional recovery following SCI [143, 144] and traumatic brain injury [145], possibly via immunomodulatory mechanisms [20]. However, not all up‐regulated ECM molecules are detrimental—some play essential roles in supporting regenerative growth [146]. For instance, inhibiting the up‐regulation of tenascin‐C significantly reduced spontaneous corticospinal axon regrowth and impaired locomotor recovery after SCI [147]. Mechanistically, tenascin‐C functions as a ligand for α9β1 integrins, and when overexpressed, modestly enhances axonal regeneration following dorsal root crush injury [148]. Paradoxically, growth‐inhibitory molecules including CSPGs and Nogo‐A at the lesion could attenuate integrin‐mediated signaling by reducing the levels of phosphorylated focal adhesion kinase (FAK) and Src [149]. This inactivation appears linked to the absence of kindlin‐1 expression in adult sensory neurons. Remarkably, overexpressing kindlin‐1 reactivated integrin signaling, enabling robust neurite outgrowth in cultured DRG neurons despite the presence of growth‐inhibitory molecules including CSPGs and Nogo‐A [150]. Overexpression of both kindlin‐1 and α9 integrin facilitated long‐distance axon regrowth and improved sensory function recovery after SCI [122], with recent network analysis suggesting this pro‐regenerative effect involved enhanced axonal transport and trafficking [123]. Indeed, integrin‐mediated axon regeneration ultimately depends on efficient axonal transport mechanisms. While efficient axonal transport of α9β1 integrin in adult sensory neurons contributes to improved regeneration of sensory axonal fibers following SCI and integrin activation, this transport is notably restricted in RGC axons: α9β1 integrin could be trafficked efficiently within the retinal RGCs, but showed extremely limited transport into RGC axons along the optic nerve [151]. Furthermore, non‐regenerative adult RGCs generally express lower levels of integrin subunits compared to highly regenerative DRG neurons, with particularly low expression of pro‐regenerative α9 integrins [124, 125]. To facilitate robust integrin‐mediated CNS regeneration, a highly coordinated machinery for effective axonal transport is therefore essential (Figure 3F). This can be achieved by reducing ARF6 activation or overexpressing PI3Kδ to efficiently deliver active integrins towards the growth cones of CNS axons [152, 153, 154]. Similarly, overexpression of protrudin enhanced the axonal transport of Rab11 vesicles and their α9 integrin cargo in cultures (Figure 3F), thereby promoting the regenerative growth of injured RGC axons following ONC [126]. More recently, a study has reported that ONC induced up‐regulation of α5β1 and α_v_β1 integrins in injured RGCs, both of which are known to interact with fibronectin [125]. Although fibronectin is not a major ECM component in the adult CNS, its expression increased in response to ONC [155]. Intravitreal injection of recombinant fibronectin containing a motif that interacts with α5β1 and α_v_β1 integrins significantly enhanced axon regeneration after ONC [125]. Further investigation is necessary to explore the potential roles of other integrins in regulating CNS regeneration, particularly within the context of ONC [156].

### 4.4 Mitochondrial Dynamics Is Crucial for Regeneration Success

Axon regeneration is an energy‐demanding process that relies on a continuous energy supply via active axonal transport of mitochondria [157]. Regenerating peripheral axons exhibit an increase in mitochondrial movement to meet the heightened energy demands of axonal elongation [158, 159]. In contrast, neurons that have poor regenerative capacity demonstrate a marked decline in mitochondrial transport [72]. Enhancing mitochondrial transport by genetic ablation of the mitochondria‐anchoring protein syntaphilin augmented energy supply to distal axons (Figure 3G), thereby promoting robust axon regeneration and functional recovery following SCI [72, 73]. Similarly, overexpression of Armcx1 in RGCs enhanced mitochondrial transport (Figure 3G) and significantly improved axon regeneration and neuronal survival after ONC [127]. Recently, we identified M1, a mitochondrial fusion promoter, as a novel pharmaceutical agent that enhanced mitochondrial transport and facilitated remarkable axon regeneration across the full length of the optic nerve. M1 treatment enabled the reinnervation of regenerated RGC axons to multiple visual targets in the brain following ONC. Notably, M1 administration alone was sufficient to restore pupillary light reflex and, in some mice, evoked responses to looming stimuli. The pro‐regenerative effects of M1 were mediated, at least in part, by its modulation of mitochondrial fusion proteins OPA1 and MFN2 (Figure 3G). Silencing either OPA1 or MFN2 in injured RGCs completely abolished the pro‐regenerative effects of M1 [36].

### 4.5 Targeting Lipid Metabolism to Boost Axon Regeneration

Emerging evidence suggests that disrupted lipid metabolism also contributes to regeneration failure in the adult CNS. Lipidomic analyses of injured RGCs and optic nerves have revealed substantial and distinctive differences in lipid composition between highly regenerative (e.g., zebrafish and *Xenopus laevis* ) and non‐regenerative species [160, 161] and across mammalian optic nerve regeneration models [162, 163]. A detailed comment on these findings has been reviewed elsewhere [164]. In cultured hippocampal (CNS) and DRG (PNS) neurons, cholesterol depletion increased growth cone size (Figure 3H), promoted the formation of filopodia, and thereby enhanced neurite outgrowth [165]. Supporting the role of cholesterol metabolism in axon regeneration, the stem cell marker Prom1 was progressively down‐regulated in DRG neurons upon maturation. Neuronal‐specific overexpression of Prom1 suppressed genes involved in cholesterol metabolism through Smad‐mediated signaling pathways (Figure 3H), facilitating accelerated axonal regrowth in injured peripheral nerves [128]. In injured RGCs, a metabolic switch from phospholipid (PL) production (essential for membrane biogenesis and axonal elongation) to triglyceride (TG) accumulation for energy storage has been observed (Figure 3H). Shortly after ONC, this aberrant shift in lipid metabolism was driven by elevated levels of two key enzymes: Lipin1, which catalyzes the conversion of phosphatidic acid (PA) to diglyceride (DG), and diglyceride acyltransferases (DGATs), which convert DG into triglyceride (TG) (Figure 3H). Deletion of Lipin1 or DGAT1/2 reversed this shift, restoring glycerolipid metabolism towards back to PL production for the formation of new membrane and thereby supporting regenerative growth after ONC [74]. Additionally, Lipin1 deletion increased levels of PA and lysophosphatidic acid (LPA) in cultured CNS neurons. PA activates both mTOR and JAK/STAT signaling, while LPA also activates JAK/STAT signaling (Figure 3H). Both PA and LPA increased neurite outgrowth in cultured neurons, largely due to the enhanced availability of PLs for membrane expansion during axon elongation [75], which eventually led to the enhanced regenerative growth following ONC and SCI [74, 75].

## 5 Regenerative Therapies for Glaucoma: A Lesson From Pre‐Clinical Studies of Optic Nerve Injury

In preclinical studies, ONC represents a relatively straightforward and highly reproducible rodent model that faithfully mimics the clinical manifestations observed in patients with traumatic optic neuropathy [166]. This model exhibits minimal spontaneous axon regeneration from axotomized RGCs, substantial neuronal loss, absence of remyelination, and irreversible visual impairment [36, 37, 167, 168]. A complete crush of the rodent optic nerve thoroughly eliminates all RGC axons distal to the lesion, with virtually no spared fibers beyond the crush site [36, 37]. Consequently, any axons growing across the lesion following treatment can be confidently identified as regenerating axons extended from surviving RGCs, which enables precise quantification of true regenerative responses, thereby providing a reliable platform for testing novel regenerative interventions aiming to stimulate axon regeneration following ONC. In contrast, glaucoma manifests as a chronic optic neuropathy frequently associated with elevated IOP. In neuroregeneration research, ONC is often employed as a surrogate model for glaucoma, presuming similar mechanisms that underlie neuronal loss and optic nerve degeneration in both conditions [129, 169]. However, compelling evidence indicates that transcriptomic changes within RGCs in response to traumatic optic nerve injury differ vastly from those observed in glaucoma [170]. Furthermore, temporal alterations in RGC activity following traumatic versus glaucomatous optic neuropathy were not identical [48]. Thus, establishing clinically relevant glaucoma models is crucial to evaluate the efficacy of various regenerative therapies in preventing RGC loss, promoting axon regeneration, and preserving visual function.

Numerous experimental pre‐clinical glaucoma rodent models have been developed over time to obstruct aqueous humor outflow from the trabecular meshwork, leading to sustained ocular hypertension. These include models based on microbead occlusion [65, 171], laser‐induced injury [172, 173], and intracameral injection of silicone oil [48, 174] or chemically cross‐linked hydrogel [175]. Each of these models reliably reproduces the progressive thinning of the ganglion cell complex (GCC) and degeneration of RGC axons as observed clinically due to sustained IOP elevation, faithfully recapitulating key pathophysiological features of human glaucomatous neurodegeneration [176].

Historically, DBA/2J mice served as a congenital experimental glaucoma model, in which elevated IOP typically develops by 6 months of age, followed by progressive optic nerve degeneration. Severe optic nerve damage is evident by 12 months, providing a substantial therapeutic window for assessing the potential neuroprotective or neuroregenerative effects of a therapeutic intervention [177]. However, a recent study has questioned the suitability of this model for pharmaceutical evaluation. Progressive deterioration in ocular structural integrity compromises accurate IOP measurement and hinders reliable functional assessments such as electroretinography (ERG), as well as structural evaluations using techniques such as optical coherence tomography (OCT) and fundus imaging. Additionally, systemic complications including cardiac calcification, thoracic cavity malformation, bladder obstruction, and aortic aneurysm significantly increase mortality over time [178]. These complications necessitate larger initial cohorts, which not only conflict with the growing emphasis of the NC3Rs strategy (Replacement, Reduction, and Refinement) in animal research [179], but also raise serious animal welfare concerns, as most mice must be maintained for extended housing periods to achieve substantial IOP elevation, leading to prolonged suffering in the majority of animals [180]. Compounding these challenges, the inherently poor breeding performance of DBA/2J mice presents an additional obstacle for researchers seeking to employ this strain as a glaucoma model [181]. Importantly, despite these limitations, DBA/2J mice remain a clinically relevant model of pigmentary glaucoma [182], as disease progression in this strain is driven by iris pigment dispersion and trabecular meshwork dysfunction [183, 184], closely mirroring the underlying etiology of pigmentary glaucoma in patients [185].

Given that glaucomatous degeneration occurs in a substantial proportion of patients with normal IOP levels (i.e., normal tension glaucoma; NTG) [186], alternative experimental models for NTG might be necessary. Recently, researchers utilized glutamate/aspartate transporter (GLAST)‐deficient mutant mice (i.e., GLAST knockout; GLAST‐KO) as an NTG model [187]. These knockout mice recapitulated the loss‐of‐function mutations in the EAAT1 gene (the human homolog of mouse GLAST) observed in NTG patients [188]. In GLAST‐KO mice, substantial RGC loss was observed as early as three weeks of age, with visual impairment documented by 12 weeks [187]. These characteristics make GLAST‐KO mice a valuable and relevant model for evaluating therapeutic strategies for NTG.

While conventional IOP‐lowering therapies (Figure 1) may merely slow the progression of glaucoma by reducing mechanical stress on the optic nerve, they fail to address the vulnerability of RGCs to degeneration, which ultimately leads to irreversible visual field deficits [14]. Consequently, there is growing interest in developing novel therapeutic strategies aimed not only at promoting RGC survival but also at rebuilding axonal projections between the retina and brain, with the ultimate goal of restoring visual function [189]. Accumulating evidence suggested that augmenting the neuronal‐intrinsic regenerative capacity of injured RGCs offered a promising approach to restore vision, as demonstrated in experimental glaucoma models. Most notably, the neurotrophic factor CNTF has long been recognized for its ability to induce long‐distance axon regeneration after ONC [190, 191, 192], and enhance RGC survival in experimental rat glaucoma models [193, 194]. While CNTF levels were substantially reduced in the affected eyes of glaucoma patients [195], sustained intraocular CNTF delivery via NT‐501 implants (developed by Neurotech Pharmaceuticals Inc.) has shown promising neuroprotective effects in early‐phase clinical trials, increasing retinal nerve fiber layer thickness and improving visual fields in glaucoma patients with an excellent safety profile [196]. This supports the development of complementary therapies that directly target RGCs for enhanced neuroprotection and regeneration [189]. NT‐501 has recently been approved by the FDA for the treatment of idiopathic macular telangiectasia type 2 in adults [197].

A recent single‐cell transcriptomic analysis of regenerating RGCs induced by PTEN deletion revealed genes critical to the regenerative phenotypes resulting from this genetic manipulation [169]. Importantly, overexpression of Anxa2, Mpp1, and Spp1 (osteopontin) alone proved sufficient not only to stimulate robust axon regeneration in ONC mice but also to preserve visual functions in a silicone oil‐induced ocular hypertension (SOHU) glaucoma mouse model [169, 198]. Remarkably, the pro‐regenerative effects of osteopontin were markedly potentiated when combined with other neurotrophic factors, including insulin‐like growth factor 1 (IGF1) and CNTF, enabling axon regeneration throughout the entire length of the optic nerve following ONC [199]. Similarly, overexpression of Lhx2 in injured RGCs promoted robust optic nerve regeneration and conferred significant neuroprotection following ONC. Lhx2 overexpression also prevented RGC loss in a microbead‐induced mouse model of glaucoma and preserved RGC function in an experimental model of NMDA‐induced excitotoxicity [112]. TrkB is a member of the neurotrophic tyrosine kinase receptor family that regulates cellular growth and survival through its interaction with brain‐derived neurotrophic factor (BDNF), which subsequently activates the Ras/PI3K/AKT signaling cascade. The BDNF/TrkB signaling axis is particularly well recognized for its essential role in supporting RGC survival with significant therapeutic implications for glaucoma [200]. A recent study demonstrated that overexpression of a constitutively active form of TrkB—achieved through farnesylation of the receptor's intracellular domain (F‐iTrkB)—enabled injured RGCs to sustain long‐distance axon regeneration across the entire optic nerve and reach the optic chiasm within four weeks post‐ONC, without the need for exogenous BDNF supplementation. More importantly, F‐iTrkB overexpression also conferred significant neuroprotection and preserved visual function in two experimental models of glaucoma: GLAST‐KO mice with normal IOP and SOHU mice with elevated IOP [110], underscoring the therapeutic potential for TrkB activation in treating both NTG and ocular hypertension‐induced glaucoma. Additionally, rejuvenating the adult RGCs using three Yamanaka transcription factors (OCT4, SOX2, and KLF4) has shown remarkable efficacy in stimulating robust axon regeneration in ONC mice via TET1‐ and TET2‐dependent mechanisms. This cocktail of gene manipulations also promoted effective neural repair and restored the lost vision in a microbead‐induced ocular hypertension model [129].

Ocular hypertension induced by either microbeads or silicone oil severely impairs axonal mitochondrial transport [130, 201]. Likewise, optineurin (OPTN) mutations are associated with NTG [202], at least partly due to a dramatic reduction in axonal mitochondria across the optic nerve leading to glaucomatous neurodegeneration. Notably, overexpression of functional OPTN alongside the mitochondrial transport complex KIF5B/TRAK1 not only induced substantial axon regeneration after ONC but also preserved visual functions in SOHU glaucoma mice [130]. We recently demonstrated that a traditional Chinese medicine, *Lycium barbarum* polysaccharides, significantly enhanced the intrinsic regenerative capacity of RGCs to enhance optic nerve regeneration following ONC [37]. This bioactive compound has long‐standing implications for glaucoma treatment, showing potent neuroprotective effects in several experimental models of glaucoma (summarized in a review [203]). Importantly, combining such regenerative approaches targeting both neuronal‐intrinsic regenerative capacity and axonal mitochondrial transport with complementary neuroprotective strategies, such as restoring endoplasmic reticulum (ER) homeostasis using maprotiline [204] or ATF4/CHOP inhibition (via genetic deletions or the pharmaceutical inhibitor ISRIB) [65, 205], or preventing nicotinamide adenine dinucleotide (NAD^+^) breakdown via SARM1 inactivation [206], presents viable options for reversing visual impairment caused by glaucomatous neurodegeneration, warranting further investigations.

## 6 Current Challenges of Regenerative Therapies and Future Directions: Bridging the Gap for Translatability From Bench to Bedside

### 6.1 Short‐Distance Axon Regeneration

**FIGURE 4:**
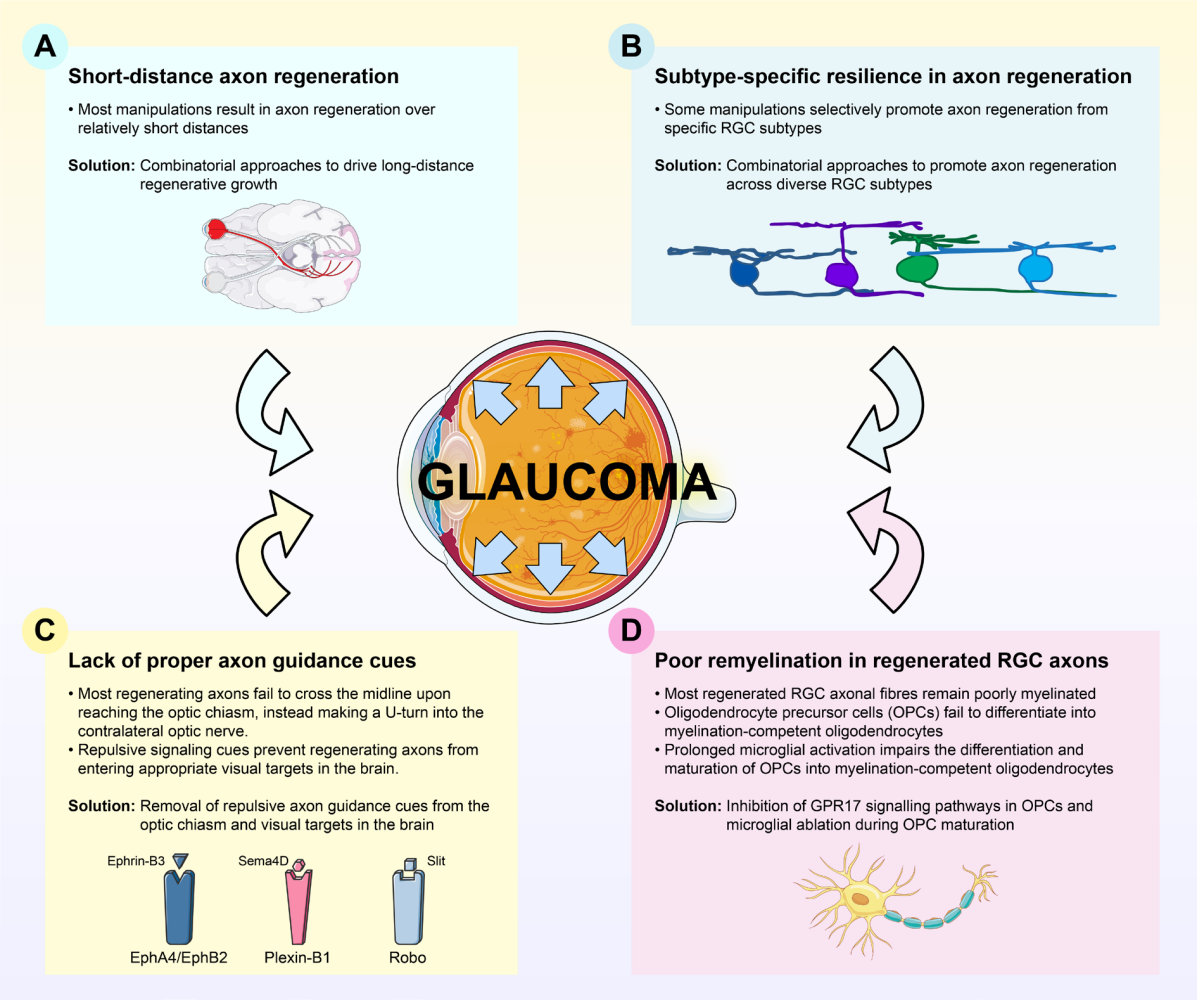
Current challenges in regenerative therapies for future glaucoma treatment and potential strategies to overcome these limitations. (A) Most gene manipulations (e.g., PTEN deletion) or pharmacological interventions (e.g., *Lycium barbarum* polysaccharide) targeting the neuronal‐intrinsic regenerative capacity of RGCs only stimulated axon regeneration over a relatively short distance. To extend regenerative growth, combinatorial approaches targeting multiple pro‐regenerative signaling pathways (e.g., PTEN/SOCS3 co‐deletion) enabled RGC axons to regenerate over long distances and enter appropriate visual targets in the brain for target reinnervation. (B) Certain gene manipulations (e.g., PTEN deletion) or pharmacological interventions (e.g., osteopontin) selectively promoted axon regeneration in specific RGC subtypes. To broaden the regenerative response, combinatorial strategies might be required to stimulate axon growth across diverse RGC populations. (C) Although some therapeutic interventions allowed RGC axons to regenerate the full length of the optic nerve, many regenerating axons fail to cross the midline at the optic chiasm. Some regenerating axons even made a U‐turn and entered the contralateral optic nerves. Repulsive molecular cues along the visual pathway prevented the entry of RGC axons into target areas for the formation of functional synapses, thereby limiting visual functional recovery. To tackle this challenge, targeted removal of these repulsive signaling cues could guide the regenerating axons towards correct pathways and thereby promote functional reinnervation. (D) In most cases, while long‐distance axon regeneration was successfully achieved, most of these regenerated RGC axons remained poorly myelinated, impairing nerve conduction and thereby limiting visual function restoration. To resolve this issue, inhibition of GPR17 signaling pathways in oligodendrocyte precursor cells (OPCs) alongside microglial ablation during OPC maturation showed promise to promote OPC differentiation into myelination‐competent oligodendrocytes. Further work is needed to identify therapeutic agents that can promote robust remyelination of regenerated RGC axons to restore visual function.

Fundamentally, restoration of visual function in glaucomatous conditions or other forms of optic neuropathy requires RGC axons not only to regenerate across the entire length of the optic nerve (approximately 3.5–5 cm in humans) [207], but also to extend into the optic tract and establish functional connections with various visual targets in the brain [92]. However, manipulations of single genes typically result in axon regeneration over relatively short distances (Figure 4A): even PTEN deletion, one of the most extensively studied interventions, induces axonal regrowth that typically ceases within 2–4 weeks post‐ONC [38, 52]. Certain interventions (e.g., PTEN/SOCS3 co‐deletion, Lin28a overexpression, myosin IIA/B knockout, M1 or glycopyrrolate treatment) have sustained long‐distance axon regeneration, enabling RGCs to extend across the entire optic nerve. Nevertheless, only a small number of axons enter the brain and successfully innervate their targets, which limits the extent of functional visual recovery [36, 37, 120].

### 6.2 Selective Axon Regeneration Among RGC Subtypes

More concerning is that some manipulations selectively promote axon regeneration from specific RGC subtypes (Figure 4B). For instance, whilst intrinsically photosensitive RGCs (ipRGCs) constitute only 5% of total RGCs, they comprise a significant proportion of regenerating axons following ONC in CNTF‐overexpressing mice [191]. ipRGCs also maintain elevated mTOR activity post‐ONC [208], partially explaining their inherently superior intrinsic regenerative capacity for spontaneous axon regeneration compared with other RGC subtypes [191]. PTEN deletion, or overexpression of osteopontin and IGF‐1, preferentially stimulates axon regeneration from α‐RGCs [133, 209], whereas exogenous administration of SDF‐1 promotes axon regeneration from RGC subtypes other than α‐RGCs [45]. Intriguingly, SOX11 overexpression drives axon regeneration in non‐α‐RGCs but simultaneously eliminates α‐RGCs following ONC [119].

Subtype‐specific vulnerability is another critical factor: whilst some RGC subtypes (e.g., α‐RGCs and ipRGCs) exhibit resilience to axotomy, others (N‐RGCs, F‐RGCs, W3‐RGCs, and ooDSGCs) are vulnerable to injury [133, 209, 210] and glaucomatous conditions [198]. Thus, combinatorial approaches (e.g., PTEN/SOCS3 deletion plus CNTF overexpression) may be necessary to promote both neuroprotection and axon regeneration across a broader range of RGC subtypes and facilitate target‐specific reinnervation and functional restoration [209].

### 6.3 Lack of Proper Axon Guidance Cues

Although certain combinatorial approaches (e.g., PTEN/SOCS3 deletion plus c‐Myc and CNTF overexpression; or PTEN deletion plus zymosan and cAMP treatment) can drive long‐distance axon regeneration [116, 211], many regenerating axons (74%) fail to cross the midline upon reaching the optic chiasm, with some (10%) making a U‐turn to grow into the contralateral optic nerve, rather than proceeding along the optic tract into the brain for target reinnervation [212]. This aberrant growth trajectory substantially reduces the number of regenerating axons reaching their proper targets to reform functional neuronal circuits, ultimately compromising functional recovery (Figure 4C). Recent studies revealed that ONC induced extensive modifications in proteomic profiles within the optic chiasm and various subcortical visual targets, including the suprachiasmatic nucleus (SCN) and lateral geniculate nucleus (LGN). In the optic chiasm, repulsive axon guidance molecules such as Ephrin‐B3 and Sema4D were highly expressed [213], and both ligands could induce growth cone collapse when interacting with their respective receptors EphA4/EphB2 and Plexin‐B1 expressed in regenerating RGC axons [214, 215]. Remarkably, silencing EphA4/EphB2 and Plexin‐B1 altered the trajectories of regenerating axons, which had been stimulated by PTEN/SOCS3 co‐deletion combined with c‐Myc and CNTF overexpression, resulting in more axons crossing the midline and extending into the optic chiasm for target re‐innervation after ONC [213]. Additionally, silencing Slit/Robo repulsive signaling directed regenerating RGC axons to enter the SCN and form functional synapses, leading to recovery of circadian activity [212]—the primary function of the SCN as the master regulator of circadian rhythms. These findings highlight the utmost importance of proper axon guidance not only for long‐distance regenerative growth but also for accurate target innervation and meaningful functional recovery.

### 6.4 Poor Remyelination in Regenerated RGC Axons

Despite viable approaches supporting long‐distance axon regeneration and formation of functional synapses in subcortical visual targets, most regenerated axonal fibers remained poorly myelinated (Figure 4D), significantly limiting visual functional recovery post‐ONC [199]. While certain therapeutic strategies induce both extensive axonal regeneration and remyelination (e.g., PTEN deletion + zymosan + cAMP analogue) [47] or promote axonal regrowth and targeted reinnervation alone (visual stimulation + Rheb1 overexpression) [92], resulting in partial restoration of visual function following ONC, numerous other approaches (e.g., osteopontin + IGF‐1 + CNTF (OIC) [199], M1 [36], and glycopyrrolate [37]) have failed to achieve substantial functional improvement due to inadequate remyelination. Following ONC, the adult optic nerve loses its capacity for myelin repair, resulting in compromised nerve conduction (evidenced by increased latency in visual‐evoked potentials) [216] and persistent visual impairment [217, 218]. Many pro‐remyelinating therapeutics have been developed in experimental models of multiple sclerosis (MS) [219]. Most notably, the potassium‐channel blocker 4‐aminopyridine (4‐AP), licensed by the FDA for improving walking in MS, could transiently enhance visual functional outcomes by boosting nerve conduction along regenerated but demyelinated axons following pro‐regenerative treatments (OIC, M1, or glycopyrrolate treatment) [36, 37, 199]. While residential oligodendrocyte precursor cells (OPCs) underwent rapid proliferation within the initial week post‐ONC, they failed to differentiate into mature myelination‐competent oligodendrocytes, partially due to sustained GPR17 activation in OPCs and prolonged microglial activation. Genetic deletion of GPR17 or pharmacological inhibition of leukotriene receptors using montelukast, combined with microglial ablation during OPC maturation, resulted in robust myelination of regenerating axons [168]. It would be of considerable interest to investigate whether pro‐remyelinating agents such as opicinumab (a monoclonal antibody targeting the remyelination inhibitor LINGO1) can promote OPC maturation and remyelination for clinically meaningful visual restoration in ONC mice when co‐administered with neuroregenerative agents [219]. Currently, opicinumab is undergoing phase 2 clinical trials for acute optic neuritis. Initial findings have been encouraging, as opicinumab modestly improved visual‐evoked potential latency in patients with acute optic neuritis [220, 221], despite no significant improvement in visual acuity or retinal structure, suggesting potential physiological benefits that warrant further investigation.

## 7 Harnessing Neural Plasticity to Support Targeted Reinnervation and Visual Recovery

**FIGURE 5:**
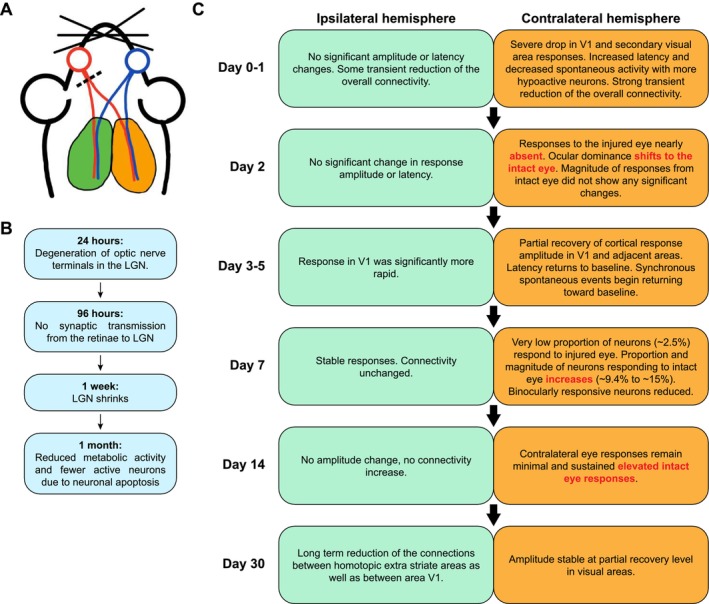
Cortical changes in the brain in response to optic nerve crush injury. (A) Schematic diagram illustrating optic nerve crush (ONC) in the left eye and the corresponding neural projections to the left and right hemispheres. Green indicates the ipsilateral hemisphere to the injured eye; orange indicates the contralateral hemisphere. (B) Timeline of degenerative changes in the visual thalamus lateral geniculate nucleus (LGN) after ONC. (C) Timeline showing the activity changes in the primary visual cortex (V1) within ipsilateral and contralateral hemispheres over time post‐ONC.

A potentially promising but comparatively underexplored strategy to promote targeted reinnervation is to harness the intrinsic plasticity of the brain (Figure 5A). By engaging residual circuitry through repetitive stimulation [222], it may be possible to restore visual functions even when structural damage to the optic nerve is substantial. Early studies in cats showed that limited neuronal plasticity occurs in the LGN of the thalamus within hours following an acute optic nerve injury at the optic disc (Figure 5B) [223, 224]. In mice, some degree of recovery of visual cortical activity was detected as early as 3–5 days post‐ONC in mice [225, 226]. More remarkably, up to 98% of the deafferented neurons developed new receptive fields within 3 months after a retinal lesion (Figure 5C) [227]. Together, these findings underscore the adaptive potential of surviving visual circuits that could be leveraged to aid functional recovery.

The interplay between neural activity and intrinsic regenerative programmes appears particularly important for successful reinnervation. Following ONC, receptivity visual stimulation coupled with elevated mTOR activity through Rheb1 overexpression facilitated long‐distance axon regeneration, aiding these regenerating axons to innervate appropriate visual targets, resulting in measurable visual functional improvement [92]. This illustrates the powerful synergy between activity‐dependent cues and intrinsic growth programmes, suggesting that manipulating neural activity can augment pro‐regenerative signaling and guide regenerating axons towards correct brain targets. Intriguingly, cortical plasticity itself is tightly regulated by ECM components. Growth‐inhibitory ECM molecules are known to restrict plasticity in the adult visual cortex [228]. Conversely, remodeling ECM components using chondroitinase‐ABC to degrade CSPG or tPA to cleave extracellular plasminogen has been shown to enhance cortical plasticity following monocular deprivation [228, 229]. Similarly, neurotrophic signaling through BDNF/TrkB and its upstream pathways is essential for restoring visual responsiveness in the visual cortex [230, 231, 232].

The cholinergic system has recently emerged as another promising modulator of visual plasticity and neuroprotection. Enhancing cholinergic signaling, particularly when paired with visual stimulation, has long been recognized as potent facilitators of cortical plasticity in the visual cortex [233]. It also confers broad protective effects on RGCs, reducing neuronal loss, improving mitochondrial function, and restoring axonal transport [234, 235, 236, 237]. Critically, oral citicoline, a metabolic precursor of acetylcholine, has been shown to preserve retinal nerve fiber layer thickness and visual function in glaucoma patients, and is currently undergoing multiple clinical trials as a glaucoma treatment [238, 239]. Collectively, these studies highlight the therapeutic potential of manipulating neural circuit plasticity and neuromodulatory systems as complementary approaches to axon regeneration. Given the convergence of molecular pathways underpinning both axon regeneration and cortical plasticity, strategies that simultaneously enhance neuronal plasticity while promoting axonal regrowth represent a particularly promising avenue for fostering functional restoration along the eye‐brain axis.

## 8 Towards Next‐Generation Regenerative Therapies in Glaucoma and Optic Neuropathies

**FIGURE 6:**
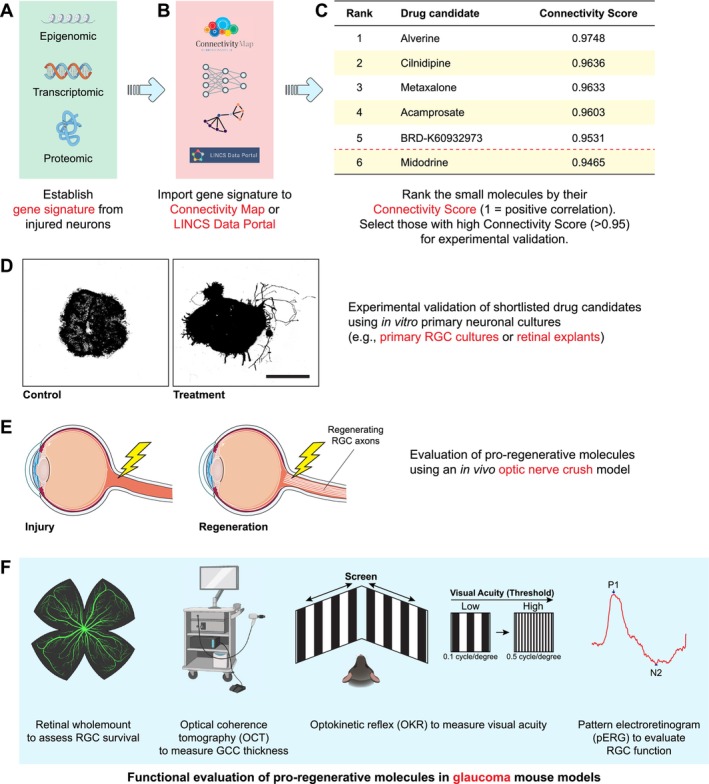
Pipeline for in silico screening of pro‐regenerative small molecules with in vitro and in vivo experimental validation. (A, B) Epigenetic, transcriptomic and/or proteomic changes in injured neurons (e.g., dorsal root ganglion (DRG) neurons, which adopt a growth‐competent state following axotomy) are served as gene signatures (A) to query against publicly available databases, such as the Connectivity Map (CMap) and LINCS (B), to identify small molecules that recapitulate similar expression profiles in neuronal cell lines following treatment of small molecules. (C) Putative pro‐regenerative small molecules are ranked according to their Connectivity Score (values approaching 1 indicate positive correlation; values approaching −1 indicate negative correlation). Those with high Connectivity Scores (typically > 0.95) are selected for experimental validation. (D) The neurite‐promoting effects of each shortlisted small molecule are assessed using in vitro primary neuronal cultures, such as primary retinal ganglion cell (RGC) cultures or retinal explant cultures as shown in the panel. Scale bar: 500 μm. (E) Molecules that demonstrate remarkable growth‐promoting effects in vitro are subsequently tested in vivo using the optic nerve crush (ONC) model, in which all RGC axons are severed. Those extending beyond the crush site represent genuinely regenerating rather than spared axons. (F) Using rodent glaucoma models, RGC survival is evaluated through retinal wholemount immunostained with pan‐RGC marker anti‐RBPMS primary antibodies and in vivo optical coherence tomography (OCT) to measure the thickness of the ganglion cell complex (GCC) following treatment with pro‐regenerative small molecules. From the same cohort of mice, visual function is assessed using the optokinetic reflex (OKR) test to objectively measure visual acuity and pattern electroretinogram (pERG) to evaluate overall RGC function.

Gene therapy is rapidly emerging as one of the most promising avenues for exploration in current vision research. A notable recent study employed CRISPR/Cas9 technology to target Aquaporin 1 expression in the ciliary body, achieving sustained reduction in IOP in an experimental glaucoma model with just a single intravitreal injection. Crucially, the adeno‐associated virus (AAV) ShH10 serotype demonstrated effective transduction in human ciliary body tissue from post‐mortem donor eyes [240], underscoring its potential to translational suitability for future clinical application. Whilst certain AAV serotypes (such as AAV2) have shown high neuronal tropism, predominantly transducing RGCs in rodent models [36, 38, 52], they also exhibit considerable expression in non‐RGC cells, including amacrine cells, bipolar cells, and Müller glia [241]. To address this limitation, a recently engineered RGC‐specific promoter restricted transgene expression exclusively to RGCs [168], enabling precise manipulation of neuronal‐intrinsic regenerative pathways. This advance opens the door to gene therapy‐based strategies that both stimulate optic nerve regeneration and confer neuroprotection in glaucoma and other optic neuropathies. Beyond gene therapy approaches, we and others have harnessed large‐scale transcriptomic datasets to screen for putative pro‐regenerative agents that recapitulate the signature transcriptomic changes in neuronal tissues. With the rapid expansion of bulk and single‐cell RNA‐sequencing (scRNA‐seq) data from injured, non‐regenerative RGCs and regenerative neurons such as DRGs, researchers can leverage these publicly accessible transcriptomic datasets (Figure 6A) to conduct in‐depth in silico drug screening. Databases such as the Connectivity Map and the Library of Integrated Network‐Based Cellular Signatures (LINCS) [242, 243, 244] (Figure 6B) enable the identification of pro‐regenerative compounds that recapitulate gene expression signatures of regenerative neurons (Figure 6C) [33, 37]. Compounds exhibiting high Connectivity Scores can subsequently be selected for experimental validation of their regenerative potential in cultured neuronal systems in vitro (e.g., primary RGC cultures or retinal explants) (Figure 6D) and within in vivo ONC models (Figure 6E). This approach has already identified drug candidates that promote robust optic nerve regeneration after ONC, including ambroxol, glycopyrrolate and mexiletine [37, 77], and agents that enhance myelin repair [245]. Further investigation is therefore warranted to assess the therapeutic potential of these pro‐regenerative small molecules in preserving visual functions in clinically relevant models of glaucoma (Figure 6F).

## 9 Conclusion

With our advanced understanding of the mechanisms underlying regenerative failure in adult retinal neurons, we are now at the forefront of developing regenerative therapies for glaucoma and other optic neuropathies. Whilst established strategies, including agents to reduce IOP in glaucoma and high‐dose steroid therapy for neuroprotection in optic neuropathies [11, 246], remain the mainstay in clinical settings, a paradigm shift should emerge towards bold, proactive interventions that harness the neuronal‐intrinsic regenerative capacity necessary for re‐building retinal‐brain connectivity and restoring visual function.

Recent advance in scRNA‐seq revealed that distinct RGC subtypes exhibited marked difference in their vulnerability to injury and intrinsic regenerative capacity, offering critical insights for targeted therapeutic development. While very few RGCs are lost during the first 3 days post‐ONC, 70% have died by the end of the first week, with this figure declining further to approximately 10% survival by 28 days post‐ONC [247]. Of 46 RGC subtypes identified, 7 subtypes (e.g., all ipRGC subtypes and 2 α‐RGC subtypes) survived robustly at 14 days post‐ONC, with some achieving survival rates of ~98%. In contrast, other subtypes (e.g., all N‐RGC subtypes) proved highly susceptible to injury, showing less than 40% survival as early as 4 days post‐ONC, and in some cases becoming almost completely depleted by 2 weeks post‐ONC [247]. This heterogeneity extends to regenerative responses: for instance, Klf9, a transcription factor known to suppress axon regeneration in ONC models [62], is expressed only in specific mature RGC subtypes [248], likely explaining why only a modest proportion of RGCs respond to pro‐regenerative effects induced by Klf9 silencing. Similarly, PTEN deletion selectively promoted axon regeneration in α‐RGCs [133, 169, 247], which comprised more than 80% of the regenerating RGC populations, whereas combining PTEN deletion with CNTF overexpression, or co‐deleting PTEN/SOCS3 with CNTF overexpression, overcame these subtype‐specific barriers to enable axon regeneration in non‐α‐RGCs [209]. Notably, certain genes (e.g., Anxa2, Plin2, Mpp1, Acaa2, Spp1, and Lgals1) were selectively up‐regulated only in regenerating RGCs following PTEN deletion, and overexpression of these genes alone was sufficient to drive robust axon regeneration after ONC, with Anxa2 and Mpp1 additionally conferring significant neuroprotection in the SOHU glaucoma model [169].

These findings underscore the need for systematic tools to predict regenerative capacity across RGC populations. The rapid expansion of scRNA‐seq resources—including retinal cell atlases from 17 species encompassing human, mouse, and non‐human primates [249], human induced pluripotent stem cell‐derived RGCs [250, 251], and experimental models of optic nerve injury [169, 209, 247, 252] and glaucoma [253]—presents an unprecedented opportunity to systematically predict and compare the regenerative potential across all RGC subtypes using a machine learning‐based “Regeneration Classifier” [254]. Network analysis of differentially expressed genes within regenerating neuronal populations has further revealed that mitochondrial bioenergetics played a pivotal role in determining regenerative success [254], positioning this cellular powerhouse as a prime therapeutic target [157]. Building upon these mechanistic insights, the convergence of artificial intelligence (AI) with drug discovery now offers exciting opportunities to systematically identify novel mitochondria‐targeted therapeutics that enhance mitochondrial functions and thereby promote regenerative growth in retinal neurons [255]. This approach enables the rapid in silico screening of vast molecular libraries to prioritize experimental validation of AI‐identified targets within established ONC and glaucoma models. Collaborative efforts that integrate expertise in ophthalmology with cutting‐edge methodologies may unlock the therapeutic breakthroughs that restore vision and benefit countless patients in the next decade.

## Author Contributions

N.P.B.A. conceived the review and formulated the focus of the review. E.B., S.E., Z.K., Y.J.S., and N.P.B.A. conducted literature search and wrote the first draft of review. N.P.B.A. and Y.J.S. edited and revised the manuscript for the final submission. All authors have read and approved the final version of manuscript for submission.

## Funding

This work was supported by Fight for Sight/Glaucoma UK (RESSGA2510); Rosetrees/Stoneygate Trust (Seedcorn2024/100044); Royal Society Project Grant (RG/R1/251126); Sight Research UK (SEE037_Au_University of Surrey) awarded to N.P.B.A., and Moorfields Eye Charity (MEC) Springboard Award (GR001652) awarded to Y.J.S.

## Conflicts of Interest

The authors declare no conflicts of interest.

## Data Availability

Data sharing not applicable to this article as no datasets were generated or analysed during the current study.
